# Pathologic expansion in the *C9orf72* gene is associated with accelerated decline of respiratory function and decreased survival in amyotrophic lateral sclerosis

**DOI:** 10.1080/07853890.2021.1896231

**Published:** 2021-09-28

**Authors:** Gabriel Miltenberger-Miltenyi, Vasco A. Conceição, Marta Gromicho, Ana Catarina Pronto-Laborinho, Susana Pinto, Mamede de Carvalho

**Affiliations:** a Physiology Institute, Instituto de Medicina Molecular, Faculty of Medicine, University of Lisbon, Portugal; b Department of Neurosciences and Mental Health, Hospital de Santa Maria-CHLN, Lisbon, Portugal

## Abstract

**Introduction:**

Respiratory insufficiency is the main cause of death in amyotrophic lateral sclerosis (ALS). As the *C9orf72* repeat expansion represents the most common genetic risk factor for this disease, we studied whether *C9orf72* modulates respiratory function and survival.

**Methods:**

Demographic and clinical data, and *C9orf72* status were collected from 372 ALS patients followed in our centre. Multiple regressions controlling for the *C9orf72* expansion, diagnosis delay, region of onset, age, gender, and comorbid frontotemporal dementia were performed to evaluate the functional and respiratory status of the patients at baseline and during disease progression – assessed using the global ALSFRS-R score and its respiratory subscore, and the predicted forced vital capacity (%FVC). A Cox regression controlling for the same variables was carried out to analyse survival.

**Results:**

At baseline, 32/372 (8.60%) patients carried the *C9orf72* repeat expansion. We found that the *C9orf72* mutation is an independent risk factor for a faster %FVC decline (*p* = .001) and shorter survival (*p* = .002).

**Conclusions:**

In ALS patients with *C9orf72* expansion, shorter survival probably derives from faster respiratory function decline. This finding may indicate a new pathogenic mechanism of *C9orf72* in ALS.

